# Robotic-assisted abdominal aortic surgery: evidence and techniques

**DOI:** 10.1007/s11701-022-01390-0

**Published:** 2022-03-04

**Authors:** René Rusch, Grischa Hoffmann, Melanie Rusch, Jochen Cremer, Rouven Berndt

**Affiliations:** 1 Clinic of Cardiovascular Surgery, University Hospital of Schleswig-Holstein, Campus Kiel, Arnold-Heller-Str. 3, Hs C, 24105 Kiel, Germany; 2 Kurt-Semm-Center for Laparoscopic and Robot-Assisted Surgery, University Hospital of Schleswig-Holstein, Kiel, Germany

**Keywords:** Robotic-assisted surgery, Minimally invasive surgery, Aortic surgery, Laparoscopy, Aorto-iliac occlusive disease, Aortic aneurysm

## Abstract

In various disciplines, robotic-assisted surgery is a well-proven routine procedure, but have never been established in vascular surgery so far. This review summarizes the results to date of robotic-assisted abdominal aortic surgery (RAAS) in the treatment of aorto-iliac occlusive disease (AIOD) and abdominal aortic aneurysm (AAA). Web-based literature search of robotic-assisted surgical procedures on the abdominal aorta and iliac arteries between 1990 and 2020 including the Cochrane Library, OVID Medline, Embase, and PubMed medical databases. All studies conducting Robotic-assisted surgery were included in the quantitative analysis regarding operative and cross-clamping times, conversion rates, mortality and morbidity within the first 30 days, and in-hospital stay. Case reports and case studies (< 5 patients) were not included. Twenty-four studies were deemed thematically eligible for inclusion; after exclusion of duplicate publications, nine met the inclusion criteria for further analysis. A total of 850 patients who had either abdominal aortic aneurysm or aorto-iliac occlusive disease underwent RAAS. One study of abdominal aortic aneurysm, three of aorto-iliac occlusive disease, and five studies of both disease entities were analyzed quantitatively. For AAA, conversion rates ranged from 13.1 to 20% and perioperative mortality ranged from 0 to 1.6% with in-hospital stay of 7 days. For aorto-iliac occlusive disease, conversion rates ranged from 0 to 20%, and perioperative mortality ranged from 0 to 3.6% with in-hospital stay of 5–8 days. RAAS has been shown to be technically feasible with acceptable short-term outcomes and questionable benefits in terms of in-hospital stay and complication rates. RAAS is currently considered only an outsider procedure. Randomized-controlled trials are indispensable for regular use in vascular surgery as well as a clear approval situation for the vascular sector.

## Introduction

Robotic-assisted systems have developed increasingly over the past 2 decades and are currently applied in various clinical indications in orthopaedics, urology, gynaecology, general and thoracic surgery [1]. This has allowed surgeons to treat complex clinical syndromes while reducing morbidity and error rates, shortening operating times and improving the overall workflow [1]. Wisselink et al. reported 2002 for the first time on robotic-assisted abdominal aortic surgery (RAAS) treating two patients with robotic-assisted aorto-iliac bypasses [2]. Both procedure and the early postoperative follow-up were without complications. In the 1990s, laparoscopic surgical techniques were developed for the treatment of infrarenal aortic aneurysm and aorto-iliac occlusive disease (AIOD) [3, 4]. In certain cases, a mini-laparotomy (hand-assisted laparoscopy) was performed for better exposure, which is still used at a few centers [5]. In the first RAAS, which featured very heterogeneous surgical techniques, only substeps, such as anastomosis placement, were performed robotically [6]. In further development, already proven laparoscopic techniques such as positioning, pneumoperitoneum, and trocar placement have been integrated into the vascular surgical procedures [3, 4]. Therefore, RAAS represent hybrid procedures combining the principles of laparoscopic surgery with the use of a robotic platform [7]. Several case reports from the 2000s demonstrate the feasibility of RAAS such as infrarenal aortic replacement and the implantation of aorto-iliac and aorto-femoral bypasses [8, 9].

The aim of this review is to critically evaluate the limited experience with RAAS so far and to describe the techniques of robotic-assisted surgery in the vascular field. In addition, due to the technical development in the last two decades and the upcoming release of new robotic platforms, we will discuss the potential applications within vascular medicine.

## Review methods

### Study selection

The web-based literature analysis examined the PubMed/MEDLINE, EMBASE, Science Direct and Cochrane databases from 1990 to 2020 for the keywords: robotic, robot, vascular surgery, aortic occlusive disease, aortic aneurysm. The search was limited to English-language publications, abstracts or conference papers were not included. The results were analyzed with regard to mortality, morbidity, and complications. In addition, the operation and cross-clamping times as well as the conversion rates to the open surgical procedure were evaluated.

### Inclusion criteria

Studies were included that contained at least five patients and described robotic-assisted procedures in the abdominal aorta and iliac arteries for aortic aneurysm or arterial occlusive disease. Only surgery that were fully robotic-assisted were considered. Because of the limited data available from comparative studies, work without a control group was also included in the analysis.

### Exclusion criteria

Duplicate publications, currently ongoing studies and non-English language publications were excluded. Case reports and case studies (< 5 patients) were also not included.

## Results

The initial search identified 253 papers from various databases, of which, after removal of thematically inappropriate papers, case series with fewer than 5 patients, and duplicates, a total of 9 publications remained that matched our inclusion and exclusion criteria. The publication years ranged from 2002 to 2016 and the sample size from 5 to 310 patients.

### Operative procedure

**Fig. 1:**
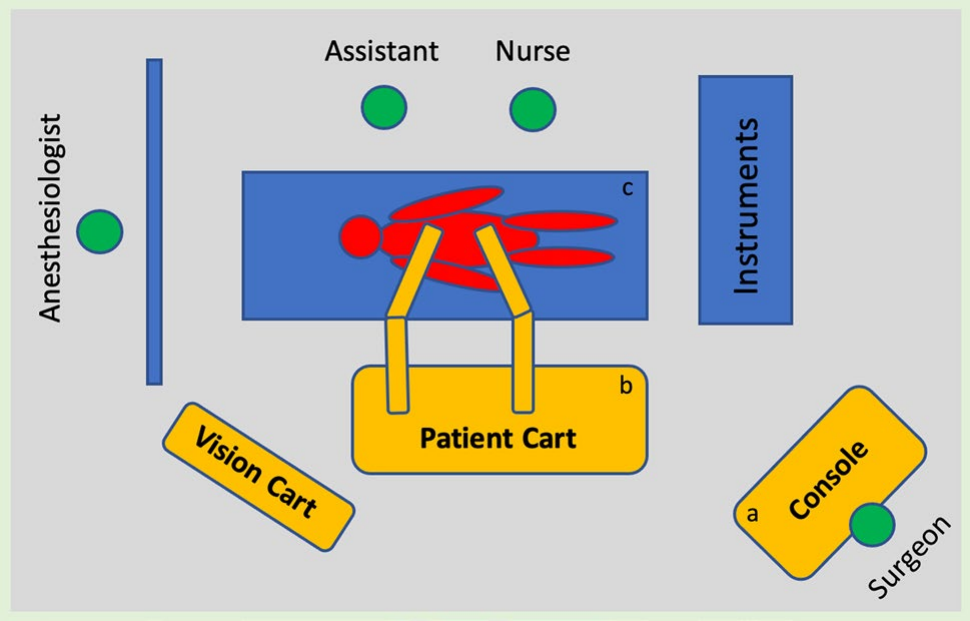
Schematic illustration of the intraoperative setup: **a** positioning of the console, **b** placement of the robotic arms, **c** patient positioning

**Fig. 2:**
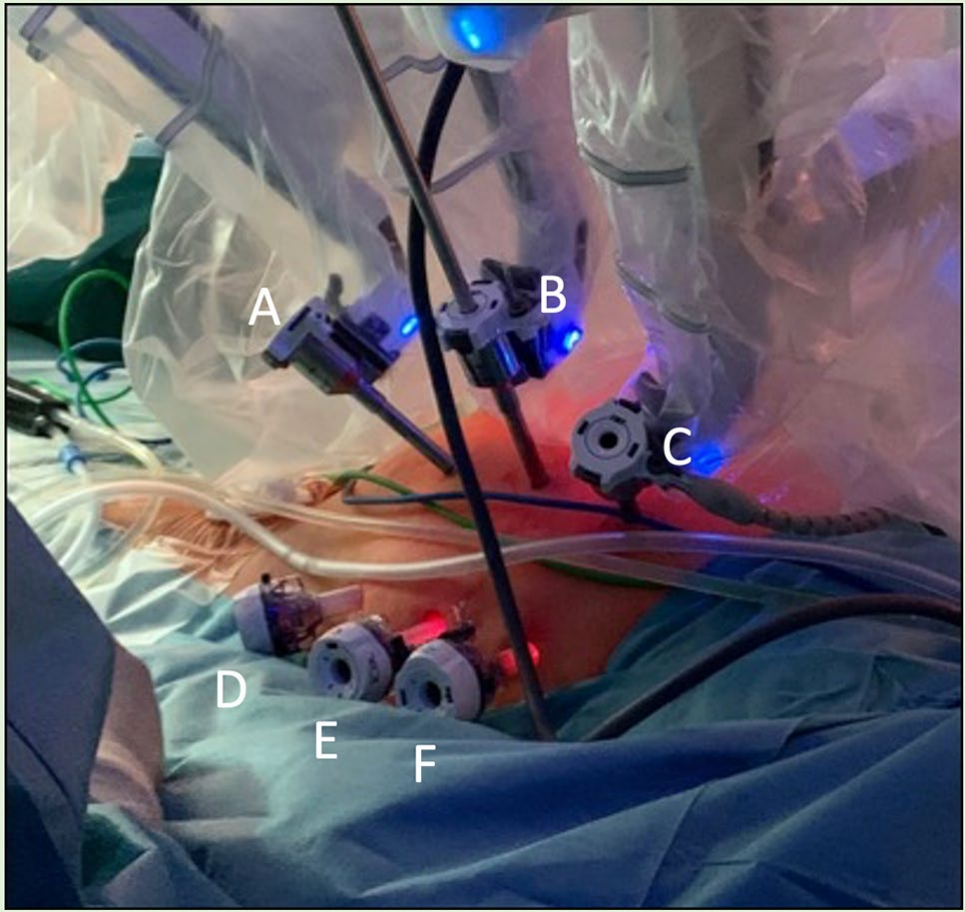
Demonstration of the robotic system in the operative setup with port placement in the abdominal region: **A** and **C** placement of the robotic arms, **B** endoscopic camera port, **D**–**F** assistant ports (Kindly provided by Prof. Stádler)

**Fig. 3:**
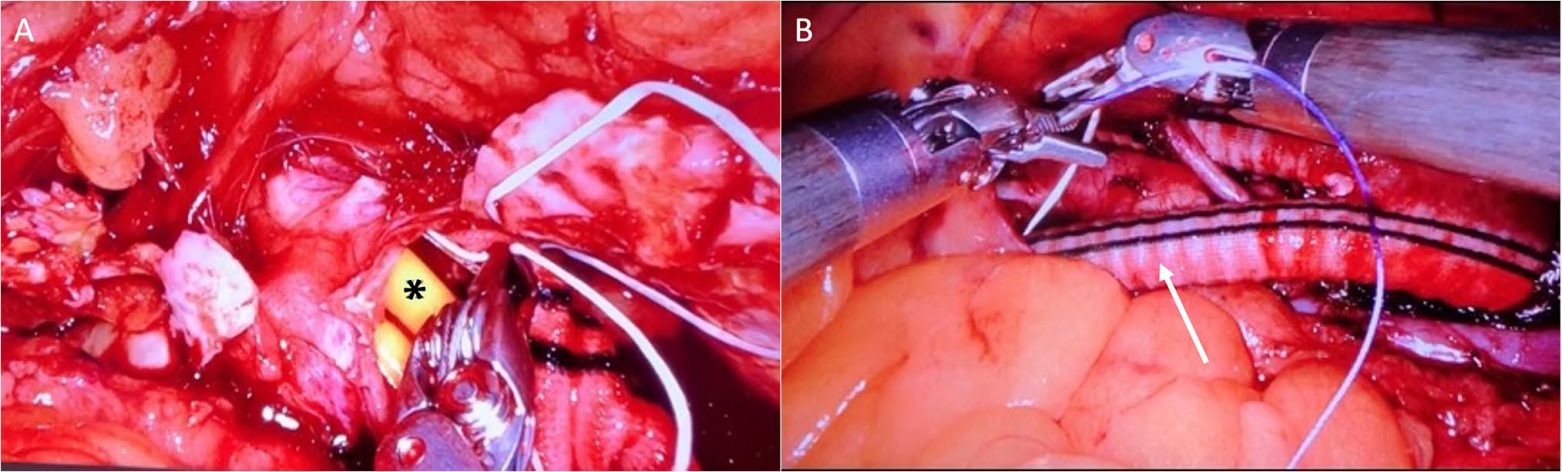
Intraoperative demonstration of robotic-assisted bypass grafting. **A** Proximal anastomosis with blocker balloons in place (black cross). **B** Distal anastomosis on the left iliac artery (with arrow) (Kindly provided by Prof. Stádler)

In reviewing the literature, only Stádler et al. presented a protocol for the application of the da Vinci system evolved from laparoscopic surgery [10, 11]. The following descriptions refer to the Prague protocol according to Stádler et al. using the da Vinci Xi^®^ robotic system (Intuitive Surgical Inc., Sunnyvale, CA, USA) [11]. The procedures were performed under general anesthesia in 30° right lateral positioning and Trendelenburg positioning (15°) with the left arm extended and the robotic components positioned on the right side of the patient (Fig. 1). At the beginning of the procedures, a pneumoperitoneum was established with insufflation of 6 l CO^2^ and an intraabdominal pressure of 12 mmHg. Six trocars were placed in two rows of three in the midclavicular line and the posterior axillary line. While the robotic instruments and the camera were inserted via the inner line, the endoscopic vascular clamps and, if required, another assist device such as a suction device were inserted via the outer trocar line (Fig. 2). The camera system was a 0- or 30-degree optic (Intuitive Surgical Inc., Sunnyvale, CA, USA). The retroperitoneal space was exposed via a modified transperitoneal approach with displacement of the abdominal organs cranially. The dorsal peritoneum was accessed longitudinally between the abdominal aorta and descending colon and fixed with transabdominal holding sutures to the front for better exposure of the aorta. Before cross-clamping the inferior mesenteric artery was ligated and lumbar arteries were clipped endoscopically. Proximal aortic cross-clamping was performed using an endoscopic aortic clamp (Storz-France, Paris, France) and distal cross-clamping was performed using either endoscopic vascular clamps or transabdominal blocker balloons (Fig. 3a). Dacron prostheses (Albograft^®^, Sorin Biomedica, Salugia, Italy) are used as vascular grafts (Fig. 3b). For the implantation of aorto-femoral bypasses, the inguinal vessels were exposed conventionally at the beginning of the procedure and the distal anastomoses are performed open-conventionally at the end of the procedure after uncoupling the robotic platform.

### Robotic-assisted surgery for aortic aneurysm and aorto-iliac occlusive disease

Kolvenbach et al. published 2004 a series of 47 patients with infrarenal aortic aneurysm treated laparoscopically, 10 of whom underwent robotic-assisted vascular anastomosis using the Zeus telemanipulator [6]. The mean operative time was 242.5 ± 40.5 min with a cross-clamping time of 95.9 ± 21.6 min. Two patients were converted to open surgery. No perioperative deaths were reported by the authors. The research group around Lin et al. (2012) showed similar results in the infrarenal aortic segment [7]. However, the patient collective comprised only six patients. The median cross-clamping time was 86 min with a median operation time of 396 ± 146 min and the conversion rate were 16.7%. The first three studies from 2008 to 2012 reported by Stádler et al. showed comparable perioperative and postoperative data with continued inclusion of patients [10, 12, 13]. The 2012 follow-up included 225 patients with different pathologies in the aorto-iliac segment [13]. The median cross-clamping time was 56 min (range 21–120 min) with a median anastomosis time of 28 min (range 12–60 min). The median operative time was 227 min (range 150–360 min) with a conversion rate of 3%. The 30-day mortality rate was 0.4%. In the largest published study by Stádler et al. in 2016, a total of 310 patients were treated in the aorto-iliac region [11]. This time a subgroup analysis was performed with regard to aortic aneurysm and aorto-iliac occlusive disease. In 61 patients with aortic aneurysm, the median operative time was 253 min (range 185–360 min) with a cross-clamping time of 93 min. The conversion rate to open surgical procedure was 13.1% with a perioperative mortality of 1.6%. The median in-hospital stay was 7 days. In comparison, in a cohort of 224 patients with aorto-iliac occlusive disease, the median operative time was 194 min (range 127–315 min) with a cross-clamping time of 37 min. The conversion rate was 0.8%. No perioperative deaths occurred and in-hospital stay had a median of 5 days. One patient had a ureteral injury after surgery, and in another case, surgery was terminated because of extensive periaortitis and was then treated by implantation of a cross-over bypass in a second operation. Two patients showed prosthesis infections in later follow-up, which were treated by surgical revision. The series of 224 robotic-assisted aorto-iliac and femoral bypasses by Stádler et al. in 2016 currently represents the largest cohort and single center experiences. According to the authors, all patients had TASC C and D lesions in the aorto-iliac region. Demographic data of the patients are not available from the publication. The authors do not specify the criteria for patient selection or preference for RAAS over endovascular or open surgery. From 2004 to 2012, 4 other papers cumulatively reported 64 robotic-assisted bypass procedures for aorto-iliac occlusive disease [7, 14–16]. In four cohorts, median operative times ranged from 188 to 493 min, cross-clamping times ranged from 60 to 86 min, 30-day mortality ranged from 0 to 5.9%, and conversion rates ranged from 0 to 20% (Table 1).

**Table 1:** Literature search on robotic-assisted surgery

Author	Year	Patients (*n*)	Pathology	System	Operation time (min)	Cross-clamping time (min)	In-hospital stay (days)	30-day mortality (%)	Conversion rate (%)
Kolvenbach et al	2004	10	AAA	Zeus	242.5 ± 40.5	95.9 ± 21.6	7.3 ± 2.4	0	20
Desgranges et al	2004	5	AIOD	da Vinci	188	75 ± 28	8 ± 2.4	0	20
Diks et al	2007	17	AIOD	Zeus/da Vinci	365 (range 225–589)	86 (range 25–205)	4 (range 3–57)	5.9	17.6
Stádler et al	2008	100	Other	da Vinci	235 (range 150–360)	42 (range 25–120)	5.1 (range 4–10)	0	3
Stádler et al	2010	150	Other	da Vinci	228 (range 150–360)	39 (range 22–120)	5 (range 4–10)	0	2.7
Novotny et al	2011	40	AIOD	da Vinci	295 (range 180–475)	60 (range 40–95)	-	0	5
Lin et al	2012	6 (AAA)	AAA	da Vinci	396 ± 146	86	7	0	16.7
		12 (AIOD)	AIOD		493 ± 36	86	7	0	0
Stádler et al	2012	225	Other	da Vinci	227 (range 150–360)	56 (range 21–120)	5 (range 4–10)	0.4	3
Stádler et al	2016	61 (AAA)	Other	da Vinci	253 (range 185–360)	93	7	1.6	13.1
		224 (AIOD)			194 (range 127–315)	37	5	0	0.8

Data are presented as: mean ± standard deviation or median with interquartile range

*AIOD:* aorto-iliac occlusive disease, *AAA:* abdominal aortic aneurysm

## Discussion

In the last 2 decades, robotic-assisted surgery has been established as an alternative to open surgery for certain indications—examples include prostatectomy and bariatric surgery [17, 18]. In the course of this development, the robotic instruments have been continuously improved, for example the single-port technique with minimized access trauma [19]. As a consequence, the range of robotic-assisted indications is continuously being expanded and allows the application to be extended to other surgical disciplines such as oral and maxillofacial surgery or pediatric surgery [20, 21].

Compared to these development, robotic-assisted surgeries could not be established in the vascular field. Initial case series published in the 2000s reported comparatively long operation and cross-clamping times and included only small numbers of patients [10, 22, 23]. At least, based on laparoscopic vascular surgery, a reproducible concept for RAAS could be developed regarding positioning, trocar placement, and transperitoneal aortic access [11]. Currently, no large randomized or comparative studies exist that present significant evidence for the use of robotic-assisted procedures in vascular surgery. To date, the research group around Stádler et al. is the only group to report notable numbers of RAAS [10–13]. The group could demonstrate comparable clamping times and operating times with shorter hospitalization in aorta-iliac pathologies compared to open surgery in 310 patients [11]. These studies do not specify clear inclusion criteria for a robotic-assisted procedures, or exclusion criteria and why no endovascular or open surgery was performed. In other surgical disciplines, robot-assisted approaches have become more common due to the minimally invasive approach [1]. In the field of vascular surgery, there is limited progress in robotic-assisted procedures, as endovascular techniques have become more important compared with open surgery, which is also reflected in the ESVS guidelines [24]. In cases of aorto-iliac occlusive disease, for example, interventional approaches have become well established in the guidelines as an alternative to open surgical procedures [25]. The treatment of infrarenal aortic aneurysms has also changed in the last 2 decades, so that in most cases, an endovascular procedure is preferred [26]. Nevertheless, open surgery still plays an important role in the treatment of AAA and AIOD, especially with regard to complication management, difficult access routes, and revision surgery.

In view of the continuing importance of open surgery, the authors believe that a critical evaluation of current and future RAAS options is necessary, despite the increasing importance of endovascular procedures. The successful long-term results of open aortic surgery combined with reduced access trauma in robotic-assisted procedures can certainly represent a positive approach in the use of RAAS. For other indications, the advantages of robotic-assisted surgery over conventional surgery in terms of smaller access trauma, lower transfusion requirements, reduced incidence of wound healing disorders, and shortened overall in-hospital stay have already been demonstrated [27, 28]. Another important aspect could be the possible reduction of incisional hernias after open aortic replacement by the minimally invasive access approach in RAAS [29]. The intuitive use of the robotic-assisted system and linear hand–eye coordination demonstrate a very sharp learning curve in the use of robotic surgical techniques [15, 30]. Another limitation of robotic-assisted vascular surgery is the absence of haptic feedback. Especially in vascular anastomosis, suture retightening is important for safe release of the anastomosis and is dependent on haptics. A combination between endovascular technique and robot-assisted surgery may be able to increase safety and reduce operative time [31]. Alternative hybrid procedures certainly represent a possibility to reduce operation times and perioperative complications. Accordingly, it is important that the surgeon undergoes an extensive training program and is aware of this limitation. Efficient simulator systems with structured training programs have also become available during the last decade [32]. The establishment of specialized training centres for robot-assisted procedures in the vascular field, similar as already existing in other disciplines, are urgently needed to reduce operation times and avoid complications [33].

Moreover, a few studies have demonstrated further potential indications for RAAS. Morelli et al. reported 2019 successful robotic-assisted elimination of type II endoleaks via clipping of lumbal arteries and inferior mesenteric artery in two patients with a maximum in-hospital stay of 3 days [34]. Considering the significant incidence of type II endoleaks of 10–25%, a minimally invasive surgical alternative seems attractive [35, 36]. In view of increasing endovascular procedures, robotic-assisted surgery represents an innovative approach, especially for patients at higher risk, which is highlighted by different research groups [34, 35, 37]. Different case reports describe the robotic-assisted approach to median arcuate ligament syndrome (MALS) [38, 39]. Podda et al. showed comparable data of robotic-assisted MALS to labaroscopic surgery [39].

The introduction and implementation of RAAS is not only challenging from a medical point of view. Up to now, robotic-assisted procedures in vascular surgery have not been specifically included in the reimbursement system worldwide and is handled as an off-label indication. It should also be mentioned that the costs of robotic-assisted surgery will be adjusted by new providers in the future, which could possibly compensate for the additional costs incurred in vascular surgery. For example, reduced in-hospital stay will certainly also play a cost-effective role, which has already been shown in the comparison of endovascular procedures to the open surgery [40]. However, for RAAS, more extensive randomized trials are still missing.

Specialized centers that use robotic-assisted systems in a multidisciplinary setting with high workloads will have an economic advantage. In addition to the financial aspects, the implementation of RAAS currently fails due to the lack in certification of the da Vinci system for the vascular sector. Therefore, RAAS can only be performed as an alternative treatment in individual cases under strict regulatory conditions. In addition to the financial aspects and the legal-regulatory requirements, the current monopoly position of the da Vinci system must also be mentioned. However, as patent rights expire, a number of new robotic platforms from various companies are about to be approved and released. Examples include the Versius^®^ (CMR Surgical), Hugo^®^ (Medtronic GmbH) and Avatera^®^ (avateramedical GmbH) systems. As a result, a change in the cost structure and also in the approval situation appears realistic in the future. To date, RAAS in the aorto-iliac segment has not been able to prevail over open and especially endovascular procedures. No randomized-controlled studies on RAAS or comparative studies on endovascular and open-operative procedures have been published so far. In addition to the increased implementation of endovascular techniques, the use of robotic-assisted procedures is limited by the fact that there is currently no approval for vascular treatment.

## Conclusions

Until now, robotic-assisted procedures do not represent an alternative to the established procedures in vascular medicine. Endovascular techniques as well as the lack of haptic feedback affect the further development of robotic-assisted surgery. The evidence of reduced surgical trauma and faster recovery with comparable operative times and results, which has already been demonstrated in other surgical disciplines, is still required for vascular surgery. It is possible that the combination of endovascular and robot-assisted techniques could increase safety and reduce suture time. In this context, minimally invasive therapy of endoleaks could represent a potential robotic-assisted approach. However, significant technical advances have essentially improved the basic requirements for RAAS in the last 2 decades. With clarification of the legal aspects, the performance of RAAS at specialized centers with robotic expertise should be feasible in the future. In this context, the use of controlled randomized trials is essential. With the implementation of new robotic platforms, continuous developments, downsizing of the systems and a new price structure can be expected.
